# The effect of extracorporeal shock wave therapy in acute traumatic spinal cord injury on motor and sensory function within 6 months post-injury: a study protocol for a two-arm three-stage adaptive, prospective, multi-center, randomized, blinded, placebo-controlled clinical trial

**DOI:** 10.1186/s13063-022-06161-8

**Published:** 2022-04-01

**Authors:** Iris Leister, Rainer Mittermayr, Georg Mattiassich, Ludwig Aigner, Thomas Haider, Lukas Machegger, Harald Kindermann, Anja Grazer-Horacek, Johannes Holfeld, Wolfgang Schaden

**Affiliations:** 1 ParaMove, SCI Research Unit, BG Trauma Center Murnau, Murnau, Germany, and Paracelus Medical University, Salzburg, Austria; 2 Spinal Cord Injury Center, Clinical Research Unit, BG Trauma Center Murnau, Murnau, Germany; 3 Institute of Molecular Regenerative Medicine, Paracelsus Medical University, Salzburg, Austria; 4 Spinal Cord Injury and Tissue Regeneration Center Salzburg (SCI-TReCS), Paracelsus Medical University, Salzburg, Austria; 5 Ludwig-Boltzmann Institute for Experimental and Clinical Traumatology, Vienna, Austria; 6 AUVA Trauma Center Meidling, Vienna, Austria; 7 Department of Orthopedics and Trauma Surgery, Klinik Diakonissen Schladming, Schladming, Austria; 8 Department of Orthopedics and Trauma Surgery, Medical University of Vienna, Vienna, Austria; 9 Division of Neuroradiology, Christian Doppler Medical Center, Paracelsus Medical University, Salzburg, Austria; 10 Department of Marketing and Electronic Business, University of Applied Sciences Upper Austria, Steyr, Austria; 11 AUVA Rehabilitation Clinic Tobelbad, Tobelbad, Austria; 12 University Clinic of Cardiac Surgery, Medical University of Innsbruck, Innsbruck, Austria

**Keywords:** Spinal cord injuries, Extracorporeal shock wave therapy, Off-label use, Treatment outcome

## Abstract

**Background:**

The pathological mechanism in acute spinal cord injury (SCI) is dual sequential: the primary mechanical lesion and the secondary injury due to a cascade of biochemical and pathological changes initiated by the primary lesion. Therapeutic approaches have focused on modulating the mechanisms of secondary injury. Despite extensive efforts in the treatment of SCI, there is yet no causal, curative treatment approach available.

Extracorporeal shock wave therapy (ESWT) has been successfully implemented in clinical use. Biological responses to therapeutic shock waves include altered metabolic activity of various cell types due to direct and indirect mechanotransduction leading to improved migration, proliferation, chemotaxis, modulation of the inflammatory response, angiogenesis, and neovascularization, thus inducing rather a regeneration than repair.

The aim of this clinical study is to investigate the effect of ESWT in humans within the first 48 h after an acute traumatic SCI, with the objective to intervene in the secondary injury phase in order to reduce the extent of neuronal loss.

**Methods:**

This two-arm three-stage adaptive, prospective, multi-center, randomized, blinded, placebo-controlled study has been initiated in July 2020, and a total of 82 patients with acute traumatic SCI will be recruited for the first stage in 15 participating hospitals as part of a two-armed three-stage adaptive trial design. The focused ESWT (energy flux density: 0.1–0.19 mJ/mm^2^, frequency: 2–5 Hz) is applied once at the level of the lesion, five segments above/below, and on the plantar surface of both feet within the first 48 h after trauma.

The degree of improvement in motor and sensory function after 6 months post-injury is the primary endpoint of the study. Secondary endpoints include routine blood chemistry parameters, the degree of spasticity, the ability to walk, urological function, quality of life, and the independence in everyday life.

**Discussion:**

The application of ESWT activates the nervous tissue regeneration involving a multitude of various biochemical and cellular events and leads to a decreased neuronal loss. ESWT might contribute to an improvement in the treatment of acute traumatic SCI in future clinical use.

**Trial registration:**

ClinicalTrials.gov NCT04474106

**Supplementary Information:**

The online version contains supplementary material available at 10.1186/s13063-022-06161-8.

## Background

Spinal cord injury (SCI) results in devastating, lifelong effects on the health and quality of life of affected patients and high financial burdens on affected individuals, their families, and the health care system in general. Beyond the primary trauma of the spinal cord, secondary health complications lead to a considerable reduction in quality of life [1].

For traumatic spinal cord injury (tSCI), an estimated global annual incidence rate of 23 per million is reported [1, 2]. In Western Europe, there are around 16 people per year and one million people who suffer from a tSCI. Globally, the incidence as well as the prevalence of tSCI is low [2].

In developed and developing countries, men between the age of 18 and 32 are most affected by tSCI. In developed countries, due to an aging population, males and females above the age of 65 are affected by falls from low altitudes (1 m or less) [2]. Age at injury has increased from 28.7 years in the 1970s to 42.2 years during 2010 to 2014 in the USA [3]. Worldwide, the mean age of patients sustaining a tSCI is reported as 33 years [4].

The leading causes of tSCI are road traffic accidents, falls, and violence. Overall, road traffic collisions are the most common cause of tSCI [3, 5]. While transport is a significant cause of SCI in all age groups, falls are the most common cause over the age of 60 [5]. Although sports-related injuries account for only 8.7% of all SCI, they represent the second most frequent cause of tSCI in those under the age of 30 years [6, 7]. Literature reports that sports with high risk of sustaining SCI are diving, skiing, rugby, horseback riding, hockey, American football, and snowboarding [1, 7]. Cervical injury is by far the most predominant level of injury for hockey (81.5% cervical), skiing (81.1%), diving (98.2%), and American football (96.3%). Over half of injuries due to horseback riding and snowboarding accidents affect the thoracic or lumbosacral neurological level [1].

One-third of patients with SCI are reported to be tetraplegic and more than 50% presenting as AIS A grade (= a complete lesion) [4, 7]. The worldwide overall sex distribution (men/women) is reported to be 3.8/1 independent of injury cause and age [4]. The proportion of cervical injuries has increased over the last decades [8].

Improvements in diagnostics, pre-hospital management, fast transfer to specialized units, good shock management, the potential for early surgical decompression, and advanced medical rehabilitation methods result in higher life expectancy and lower mortality rates in high-income countries [5, 9]. Though, life expectancy after SCI is reduced compared to the general population. The mortality depends on the level and severity of the lesion and the availability of clinical care. Mortality is particularly high in the first year after injury [5].

Despite worldwide intensive efforts, SCI remains a devastating condition for which there is yet no causal treatment available [10].

### Pathophysiology

Based on imaging and histology of the injured human spinal cord, tSCI is classified as a “contusion with cavity formation,” “massive compression,” or “laceration” [10]. The primary injury mechanism involves the initial mechanical damage due to local deformation and energy transformation that occurs within the spinal cord at the moment of injury, which is irreversible [11]. This primary mechanical injury leads to a cascade of biological events, described as “secondary injury,” which occurs within minutes to weeks after the injury and further compromises neurological function [10].

Injuries to nervous tissue are followed by a rapid process of degenerative events called Wallerian degeneration in anterograde direction from the initial injury site [12, 13]. Wallerian degeneration as one of the initial pathological results of axotomy is considered an unavoidable consequence of SCI [14]. In the central and peripheral nervous system, the early phase of Wallerian degeneration includes the granular disintegration of the cytoskeleton, in which the cytoskeletal proteins of the axon are rapidly degraded to granular residues. This occurs between 18 and 48 h after injury and progresses away from the lesion site [13]. The proximal nerve stump retrograde from the initial lesion is also affected and chromatolysis reaches up to the first Ranvier node [12]. Numerous biochemical mechanisms that might explain the progressive post-traumatic damage of spinal cord tissue have been postulated: vascular changes including hemorrhage, vasospasm, thrombosis, loss of autoregulation, breakdown of blood-brain barrier, and infiltration of inflammatory cells leading to edema, necrosis, and ischemia; free radical formation and lipid peroxidation that cause oxidative death in spinal cord neurons; disruption of ionic balance; glutamate excitotoxicity; apoptosis; and an inflammatory response following the trauma. Subsuming, the secondary injury leads to neurological impairments in both anterograde and retrograde directions owing to white matter demyelination, gray matter dissolution, connective tissue deposition, and glial scar formation. The glial scar acts like a physical barrier, preventing axons to grow through it [10].

In addition to loss of sensory and motor function, central neuropathic pain in many cases is a consequence of SCI due to maladaptive synaptic circuits in the spinal dorsal horn that result in neuronal hyperexcitability. Pain transmission in the spinal dorsal horn is persistently increased due to neuronal hyperexcitability. These neurochemical and neuroanatomical changes often elicit chronic pain syndromes after SCI [15, 16].

It has been postulated that mechanisms of secondary injury are preventable and potentially reversible [9–11].

### Extracorporeal shock wave therapy (ESWT)

Shock waves are short-term acoustic pressure pulses that travel through tissue in a wave-like manner. Shock waves can be produced by electrohydraulic, electromagnetic, and piezo-electric sources. Extracorporeal shock wave therapy is subdivided into the categories “high-energy” (for bone tissue) and “low-energy” (for soft tissue) according to the energy flux density measured in mJ/mm^2^. Another classification is “focused” or “de-focused” depending on the reflector shape. Defocused shock wave applicators provide a larger diameter of the shock wave field and therefore enable the treatment of a larger tissue area. Focused shock waves allow for concentration of energy at one small therapeutic area of interest and are typically used for lithotripsy and to foster bone healing [12, 17].

The physical impact of shock waves including pressure, tensile, and shearing forces are translated to complex biochemical signals. This signaling process is called mechanotransduction. The underlying mechanism of mechanotransduction is that organ formation and tissue regeneration is the dynamic interaction between a cell and its microenvironment, including forces applied to them [12, 18].

Several biological responses to therapeutic shock waves have been postulated, including angiogenesis and neovascularization, anti-inflammatory and antimicrobial effects, the release of growth factors, the activation of mesenchymal stem cells, stimulated cell proliferation and differentiation, and suppression of nociception [12, 18–20].

ESWT has been clinically implemented in the treatment of chronic wounds, venous or diabetic ulcers, and ischemic heart disease and to stimulate and reactivate bone healing in non-healing fractures and the integration of skin grafts [12, 17, 19, 21].

In December 2018, STORZ MEDICAL has received the CE approval for transcranial pulse stimulation for the treatment of patients with Alzheimer’s disease. This technology allows a focused stimulation of the brain at a depth of up to 5 cm [22].

### Pre-clinical and clinical data on the effect of ESWT on nerve regeneration

Hausner and colleagues found significantly greater numbers of myelinated fibers in peripheral nerves after a one-time ESWT application in an experimental model on rats after a homotopic nerve autograft into the sciatic nerve [23]. Another study by Lobenwein et al. performed a spinal cord ischemia model in mice. ESWT was applied immediately after surgery and the treated animals showed a significantly better motor function and decreased neuronal degeneration compared to the control group within the first 7 days after surgery. In the same study, ESWT was applied to human spinal cord slices ex vivo 2 h post-mortem. They found significantly higher numbers of cells in the treatment group at 24 and 48 h ﻿after treatment compared to the control slices [24]. Furthermore, low-energy ESWT for the duration of 3 weeks on a spinal cord contusion injury model in rats resulted in significantly better locomotor improvement, reduced neuronal loss, an increased vascular endothelial growth factor (VEGF) expression in various neural cells, and an upregulation of brain-derived neurotrophic factor (BDNF) compared to the control animals [25–27].

Also, several other indications for ESWT had been evaluated: Lohse-Busch et al. conducted a study on the effect of focused transcranial ESWT in patients with unresponsive wakefulness syndrome (apallic syndrome). The ESWT was applied to the neurocranium three times a week for 4 weeks with an energy flow density between 0.2 and 0.3 mJ/mm^2^. An improvement of 135.9% in the German Coma Remission Scale (KRS) and 43.6% in the Glasgow Coma Scale (GCS) was observed after four to eight (average of 4.5) treatment series. No adverse effects and no triggering of epileptic seizures have been reported [28].

Davis et al. found significantly reduced inflammatory cell infiltrate of both neutrophils and macrophages in ESWT-treated burn wounds in a murine burn wound model 24 h following burn injury. ESWT was applied once 1 h after the burn injury and resulted in a significant downregulation of the expression of several acute-phase pro-inflammatory cytokines [29].

Additionally in vitro experiments showed that ESWT significantly enhanced the expression of the anti-inflammatory cytokine IL-10 when applied to human umbilical vein endothelial cells or monocyte-derived macrophages [30, 31]. Also, shock waves did not induce activation of resting macrophages at any energy level used [31].

Summarizing these results, several in vitro studies and animal trials reported that low-energy ESWT reduces the expression of pro-inflammatory cytokines and promotes the acquisition of anti-inflammatory cytokines shortly after the application of ESWT [29–31].

Therefore, it can be hypothesized that a one-time ESWT modulates the inflammatory response in several tissues when applied within the acute phase of injury [29, 30, 32]. Also, no neural tissue damage, such as hemorrhage or vacuole formation, was observed in histological analysis after the application of low-energy ESWT to the spinal cord in a spinal cord contusion model in rats [25].

### Extracorporeal shockwave therapy (ESWT) in patients with chronic complete paraplegia (AIS A) at the thoracic level

In 2015, recruitment for a multi-center prospective randomized double-blind clinical study was started including patients suffering from chronic paraplegia (lesion between THII and THX, AIS A = complete central lesion) at least for 1 year after the initial trauma without spontaneous recovery within the last 6 months. In general, a spontaneous recovery of the senso-motoric function is decreasing with the time from injury and is therefore not expected in the study population. In these cases, treatment options are mainly limited to the improvement of the quality of life.

Currently, 34 patients from the intended 50 have been included in the study and recruitment is ongoing until the end of 2020. Therefore, results are not available yet. Until now, no severe side effect or adverse effect occurred to the study participants. Besides the multi-parametric evaluation (neurological, neurophysiological, clinical as well as functional), blood samples have been collected to multiplex protein assessment miRNA analysis.

### Safety and efficacy study: shock wave therapy in patients with ischemic spinal cord injury

Holfeld and colleagues are working on an ongoing controlled, multi-center, open trial assessing the safety and efficacy of extracorporeal shock wave therapy in patients with ischemic spinal cord injury after aortic surgery or endovascular aortic repair. The study started in July 2017. Until now, five patients have been included in the study and no severe side effect or adverse effect occurred to the study participants.

### Pilot study: the effect of ESWT on peripheral nerve regeneration

Hausner et al. are currently working on a prospective randomized double-blind pilot study in which the authors investigate the effect of ESWT on nerve regeneration after acute traumatic complete dissection of either the median or ulnar nerve and subsequent microsurgical nerve co-adaption. Until now, nine patients have been included in the clinical trial and no severe side effect or adverse effect due to the ESWT occurred to the study participants.

### Risks and benefit

Previous literature reports, that in the planned energy flux density and pulse range, no therapy-associated tissue damage has been observed neither in neural tissue nor in muscle/tendon tissue — even with repetitive application [17, 28, 33, 34].

We consider the application of low-energy ESWT after acute traumatic SCI ethically justifiable because previous studies have shown no adverse events and a positive impact on the central and the peripheral nervous system [12, 17, 23, 25, 34]. The risk to potential benefit ratio is considered in favor of the patient’s benefit.

### Study objectives

#### Primary objective

The primary aim of this study is to investigate whether a greater improvement in motor and sensory function (the AIS grade) can be achieved in patients after spinal trauma (AIS A–D) by applying a single extracorporeal shockwave therapy compared to the control group.

The primary endpoint is the degree of motor and sensory impairment in the American Spinal Injury Association (ASIA) Impairment Scale (AIS) and the International Standards for Neurological Classification of Spinal Cord Injury (ISNCSCI) Score.

The results are compared over the course of time in both groups between the treatment group (one-time low-energy focused ESWT) and the control group (placebo-ESWT).

#### Secondary objective

The secondary objectives include the assessment of changes in spasticity, the ability to walk, urological function, quality of life, and independence in everyday life over time and in comparison between the groups.

## Methods

This is a two-arm three-stage adaptive, prospective, multi-center, randomized, blinded, placebo-controlled clinical trial according to the MPG (Medizinproduktegesetz [Medical Devices Act]) which is designed as a two-arm three-stage adaptive trial.

Fourteen treating hospitals and three rehabilitation centers in Austria and Germany will participate. The allocation of the study groups will occur by means of a block randomization, in order to guarantee the best possible balance between the treatment group, the control group, and the treating hospitals. The study groups are comprised of a treatment group (extracorporeal shock wave therapy by means of electrohydraulic shock wave generation) and a control group (placebo ESWT).

To gain as homogenous groups as possible, a stratified block randomization with a block size of four will be done. The assignment to treatment and placebo group will be made in a ratio of 1:1. For stratification, three different neurological levels of injury will be used:

1. Cervical injuries: C1–C8
2. Thoracic and lumbar injuries (T1–L1)
3. Peripheral nervous system injuries (cauda equina and conus medullaris syndromes [35]; L2–S5)

Recruitment of patients takes place continually at admission to acute care in one of the 14 participating hospitals (13 in Austria, 1 in Germany). Patients and assessors of study relevant data are blinded to group allocation. The operating room staff and persons involved in the application of the study intervention are not blinded to group allocation and are required to keep it as a secret.

### Study endpoints

#### Primary endpoint

It is assumed that patients who suffer acute SCI regain more of their motor and sensory functions because of treatment with EWST than patients in the control group. Six months after the baseline measurement, patients will be invited for a follow-up visit as soon as possible. The primary endpoint will be a composite endpoint composed of the ISNCSCI subscores — motor and sensory function — to achieve the required power of the study with the expected incidence. These subscores and all secondary endpoints will be analyzed descriptively to provide further insight or to derive hypotheses as appropriate.

The primary composite endpoint is calculated as the difference between the upper and lower extremities motor score (ULMS + LLMS), light touch (LT), and pin prick (PP) at two points in time.

delta.TMSC = TMSC_after 6 months_ minus TMSC_at baseline_

TMSC = Total motor and sensory score

#### Secondary (exploratory) endpoints and control variables

- AIS classification
- Degree of spasticity according to Penn Spasm Frequency Scale
- Walking ability
- WISCI II (Walking Index for Spinal Cord Injury II)
- TUG (Timed Up and Go Test)
- 10-m timed walk
- 6-min walk test
- Urological function
- Hand motor function
- Plantar reflex
- Independence in everyday life of patients is assessed with the Spinal Cord Independence Measure (SCIM II)
- The number of study-related adverse events (AEs) are measured according to NCI CTCAE, version 5.0

### Study population

Included are female and male patients with an acute spinal cord lesion regardless of the neurological level of the lesion. Patients with either complete (AIS A) or incomplete (AIS B–D) lesion are included. A minimum age of 18 years must be reached at enrollment. No other age limit was defined. A gender distribution in favor of male patients is expected due to the higher incidence (men/women = 3.8/1) of traumatic SCI [4].

#### Inclusion criteria

- Patients with acute traumatic spinal injuries who are awake, responsive, and oriented at admission
- Patients from the age of 18 years
- Admission to hospital within 24 h after injury
- Written consent to participate in the study
- Participation in the Austrian Spinal Cord Injury Study (ASCIS)-Registry (only for the Austrian hospitals)

#### Exclusion criteria

- Serious traumatic brain injuries that prevent accurate participation in study procedures and/or adequacy of informed consent
- Participation in other interventional clinical trials
- Serious concomitant injuries that prevent the neurological initial assessment
- Pre-existing neurological conditions that affect the primary endpoint of the study and potentially mask or reduce the therapeutic effect of the ESWT application
- High-dose administration of corticosteroids such as methylprednisolone, dexamethasone, etc.
- Complete spinal cord transection
- Patients with pacemakers or implantable defibrillators
- Patients who are using devices which are sensitive to electromagnetic radiation
- (potential) Pregnancy
- Patients with tumors
- Patients with severe coagulation disorders

### Study procedures

First, eligibility for study participation will be assessed and informed consent will be obtained in a pre-screening. Additionally, a pregnancy test will be performed in women with childbearing potential prior to the study intervention. After that, eligible patients undergo a baseline examination comprising a short medical history, a neurological examination (AIS/ISNCSCI), radiological imaging, assessment of the presence of pathological reflexes (priapism, plantar reflex), and documentation of pre-operative interventions.

ESWT is applied peri-operatively under general anesthesia (ideally post-operative immediately after skin closure) or within the first 48 h after trauma.

After the study intervention, surgical interventions and intra-operative diagnosis shall be documented. Also, findings from post-operative imaging and the time sequence of the acute care chain are recorded.

The first follow-up exam should be performed as early as 24–72 h after surgery consisting of a comprehensive medical history and drug anamnesis, an assessment of pre-existing neurological deficits, a neurological examination (AIS/ISNCSCI and plantar reflex), a gait analysis, assessment of urological function, rating of injury severity (Injury Severity Score (ISS)), survey of comorbidities (Charlson Comorbidity Index (CCI)), and questionnaires addressing activities of daily life (Spinal Cord Independence Measure (SCIM) and the Walking Index for Spinal Cord Injury II (WISCI II)). Also, a patient diary is handed out to every patient during the first study visit in which patients should record spasms (Penn Spasm Frequency Scale), medication (drug name and dosage), and any important subjective or objective changes in the neurological symptoms.

The 2nd (day 14–21), 3rd (3 months), and 4th (6 months) follow-up visits consist of a neurological examination, a gait analysis, assessment of urological function, and questionnaires addressing activities of daily life. Parameters from routine blood chemistry and changes in medication shall be documented at each study visit. At the last follow-up exam (6 months), the stability of osteosynthesis is re-evaluated according to X-ray/CT, and a copy of the patient diary is filed at the study site.

The study procedures are depicted in a flowchart, as well as in tabular format in the supplementary material.

### Study assessments

#### Concomitant medication

Any medication is documented as part of standard clinical care including dose, duration, and mode of administration. Patients are encouraged to continue their medication after discharge in the prescribed form. If there is a change during the 6-month observation period, this has to be noted in the patient diary, stating the name of the drug, the dosage, and the duration of the intake.

In the study visits 5–8 (follow-up exams 1–4), the following categories of drugs are assessed: NSAIDs, antidepressants, anticonvulsants, opioids, spasmolytics, sedative-hypnotics, neuroleptics, and anticoagulants including drug name and dosage.

The administration of corticosteroids in acute spinal cord trauma is controversial and the indication is discussed in the literature [10, 36–39]. If physicians decide to administer corticosteroids in high dosage, participation in this study is no longer possible for those patients.

No additional medication is administered within the study.

#### Patient diary

The patient diary is checked by the examiner during each study visit and reviewed and discussed with the patients. The patient diary is handed out to every patient during the first post-operative days at the first study visit. Patients are required to record the following variables: spasms by spasticity rating according to Penn Spasm Frequency Scale, medication (drug name and dosage), and any important subjective or objective changes in the neurological symptoms. The item spasms shall be documented on a weekly basis immediately after receiving the patient diary. Changes in medication or important events shall be documented depending on their occurrence after discharge from the hospital. All entries shall be dated.

#### Blood samples

Blood sample collection is optional at the baseline examination. If a blood sample has been taken at baseline, it is mandatory at every other follow-up visit. If no blood sample has been collected at baseline, no further blood samples are intended.

The blood tube (one 8-ml serum tube) is cooled after removal at 4 °C and then centrifuged at 3500*g* for about 15 min and then frozen to −80 °C. Parameters to be analyzed are pro-inflammatory and anti-inflammatory cytokines and proteins (some examples are S100b, IL-6, GFAP, NSE, tau, and pNF-H) and serological biomarkers which are discovered and published during the time course of the study.

Blood sample collection will only take place in those hospitals where technical feasibility is given (−80° freezer and a centrifuge).

Additionally, parameters from routine blood chemistry shall be documented in the eCRF at baseline, the 1st, 2nd, and 3rd follow-up exam. The following markers will be included in the eCRF: WBC, lymphocytes, eosinophils, PLT, MCHC, MCH, MCV, albumin, ALP, creatinine, bilirubin, uric acid, calcium, chloride, CO_2_ content, and sodium.

#### AIS/ISNCSCI

The neurological level of injury and injury completeness will be assessed according to the American Spinal Injury Association (ASIA) International Standards for Neurological Classification of SCI (ISNCSCI). The classification system contains three elements: ASIA Impairment Scale (AIS A–E), motor score (based on the neurological examination of muscle function), and sensory score (based on the neurological examination of sensory function) [5, 40–43].

#### Spasticity assessment

The degree of spasticity will be assessed with the self-rated Penn Spasm Frequency Scale (PSFS) [44–46] on a weekly basis in the patient diary.

We refrained from a clinically rated assessment of spasticity because the level of spasticity is known to vary over time; thus, a single clinical assessment will not necessarily reflect accurately an individual’s overall level of spasticity. The principal clinical outcome measure for spasticity has been the long-established Modified Ashworth Scale (MAS) [47, 48]. Previous literature investigating the MAS reported poor inter-rater reliability [49–52] and a poor correlation with self-rated assessments of spasticity [53]. Joint contractions further decrease the reliability of the MAS [52]. Furthermore, the MAS does not account for the dependence of the resistance to the velocity of the movement and therefore does not comply with the concept of spasticity [54].

#### Gait analysis

The following scores/gait tests are assessed in patients who regain walking ability with or without auxiliaries:

- WISCI II (Walking Index for Spinal Cord Injury II)
- TUG (Timed Up and Go Test)
- 10-m timed walk
- 6-min walk test

#### Urological function

Urological function will be assessed with a query including questions regarding bladder and bowel function (for a detailed list of questions, please refer to the supplementary material).

#### Hand motor function

An evaluation of hand motor function is assessed in those patients who have their lesions above the level T5 with the Nine-Hole Peg Test (NHPT) [55], if feasible, and the Grasp and Release Test (GRT) [56]. Additionally, several grasp tasks are recorded (see supplementary material).

#### Differentiation: nerve root lesion vs. spinal cord lesion

In order to differentiate between isolated nerve root lesions and spinal cord lesions, or a combination of both, a neurologist will clinically classify the location of the lesion.

#### Charlson Comorbidity Index (CCI)

Comorbidities are quantified with the CCI once during the inpatient stay within the first days. The CCI serves as a possible explanatory variable in statistical analysis.

#### Injury Severity Score (ISS)

The Injury Severity Score (ISS) is recorded once during the inpatient stay (ideally pre-operatively; if this is not possible for reasons of timing, within the first 3 days) and serves as an explanatory variable.

#### Subjective physical activity prior to injury

Physical activity prior to the injury is assessed retrospectively at the second follow-up examination (days 14–21) using a subjective self-administered questionnaire. The level of physical activity is considered an important confounder on rehabilitation. The questionnaire has been compiled exclusively for this use case and this patient population. Different types of sports are listed, and the patients should give an estimation on how often, how long, and at what intensity they used to participate in sports.

#### Imaging

The radiological examination by X-ray and/or CT and/or MRI is carried out as part of standard clinical care and data are used for study-related purposes as well. Spinal fractures are graded according to AO classification of the spine (AO 50-53) [57–59].

If fracture classification is not possible with data from the CT scan, an MRI is recommended and the following items should be assessed: (i) spinal lesion, (ii) bone marrow edema, and (iii) ligamentous lesion. For the evaluation of spinal lesions, the MR classification system based on axial images for cervical compressive myelopathy will be used [60]. Ligamentous lesions will be quantified by assessment of the integrity of the posterior ligament complex [61–63]. Data from the MRI scan should be stored on a CD and sent to the Austrian Spinal Cord Injury Study (ASCIS) office in Salzburg, where a trained neuroradiologist will evaluate the images. This should provide a deeper insight in the prognostic value of fracture morphology.

An MRI is mandatory as early as possible in cases suspicious for complete transactional medulla lesions according to neurological examination. In addition to the standard spinal imaging, a diffusion tensor imaging (DTI) medium resolution sequence with 3 b values is recommended.

### Investigational Medical Device (IMD)

The Medical Device used in this trial is a shockwave generator produced by MTS Medical UG, 78467 Konstanz, Germany. The orthogold100® uses patented MTS Spark Wave® technology.

#### Mode of administration

The extracorporeal shockwave therapy is applied once at the level of the lesion and 5 segments above and below or below the occiput (in lesions higher than C6) and above the sacrum (in lesions lower than T12). The transducer is held in an approximated angle of 45° paravertebral from both sides. In addition, the ESWT is applied to the soles of both feet on the medial side of the plantar surface. In patients who require surgical treatment, the ESWT is applied peri-operatively under extension (about 15 to 20 min) of the general anesthesia (ideally post-operative immediately after skin closure) or within the first 48 h after trauma.

If no surgery is required, the ESWT or placebo application is applied as soon as possible within 48 h from the first recognition of neurological symptoms, both in the intensive care unit after surgery or in the normal ward.

For the application, a sterile film is placed in the treatment area and ultrasound gel is used. The study intervention takes approximately 20 min. In the control group, the same procedure is performed, but without the device emitting extracorporeal shock waves using a dummy head.

The application of the ESWT, as well as placebo ESWT, takes place in a prone position if surgical treatment takes place in a prone position. If the surgery takes place in a supine position, patients are placed in a lateral or prone position post-operatively to apply ESWT.

The treating physician together with the team of medical staff will decide whether an additional 20 min of anesthesia is justifiable for the individual patient. For those who cannot give consent to participate prior to the surgical intervention and are still eligible post-operatively (still within the time frame of 48 h), the medical team has to weigh up if a change of position to prone or lateral position is possible without compromising the individual medical condition.

The follow-up examinations shall be blinded [64]. In order to ensure blinding of the examiner, the entire operating room staff and all persons involved in the application of the study intervention are required to keep the group allocation as a secret.

#### Parameters of the ESWT

- ESWT head: focused (OE50)
- Level of filling of the therapy head: 1 (1-5)
- Frequency: 5 (3-5) Hz
- Energy flux density: 0.19 (0.15–0.23) mJ/mm^2^
- Measurement of the distance between skin surface and spinal cord at the level of the lesion according to CT to determine the pressure during treatment in order to reach the spinal cord sufficiently
- Number of impulses: 5000 at the level of the lesion (2500 pulses bilaterally paravertebral) and 1000 on the medial side of the plantar surface on both feet (500 pulses per foot)
- Area of treatment: 2500 pulses bilaterally paravertebral and 4 segments above and below (3–5 segments: 3 segments for focal lesions and 5 segments for extensive lesions)

#### Placebo, comparator

For patients randomized into the control group, a dummy applicator will be connected to the shockwave generator.

#### Blinding and unblinding

The study will be blinded for treatment so that the patient and the assessor of the neurological assessment and gait analyses are unaware of the treatment group assigned. The randomization list will be opened after the study database has been locked.

### Statistics

#### Study sample size

Sample size calculation is usually based on an effect size expected from a given treatment. Due to a lack of knowledge about the effects of EWST on sensory (SF) and motor functions (MF), such an estimation is limited. For this reason, a so-called adaptive design was chosen, corresponding to the “multi-arm multi-level approach” (MAMS approach) proposed by Jaki et al. [65]. The advantage of this design is that the effect size needed to calculate the sample size is not based on specific values, but on probabilities (*p* and p0) that can be used to identify the desired or the uninteresting effect. The probability *p* = 0.6 chosen for the study for the desired effect means that a randomly selected person in the treatment group has a 60% probability of achieving a better result than a randomly selected person in the control group. The uninteresting effect is assumed with a probability of p0 = 0.5. This value means that both the novel treatment and the placebo treatment perform equally well (Jaki et al., 2019). Another advantage of MAMS is that this form of sampling does not require any knowledge of the variance of the endpoint variables. All the calculations required for this are implemented in an R package and published accordingly (Jaki et al., 2019).

A two-armed, three-stage adaptive study design is envisaged at Neurowave. In stage 1 and stage 2, interim evaluations are carried out to determine whether the study can already be terminated. This will be the case if either the intended and thus desired result can already be sufficiently confirmed, or it becomes apparent that the study will not be a success. These interim evaluations offer the opportunity to shorten the study. If none of these two cases occur, the study will continue up to stage 3. The overarching advantage of this design is that the smallest possible number of patients can be found to demonstrate a medically relevant effect.

With a one-sided error rate of 5% and a planned power of 80%, the following design parameters could be calculated. For the upper and lower limits, the triangle test is selected [65].

**Table:** 

	Stage 1	Stage 2	Stage 3
Cumulative sample size (control):	41	82	123
Cumulative sample size (treatment):	41	82	123
Maximum total sample size: **246**			
Upper bound	2.13	1.89	1.85
Lower bound	0.00	1.13	1.85
*p*-value for H1 ((stopping for) success)	0.018	0.031	0.033
*p*-value for H0 (stopping for a lack of efficacy)	0.500	0.130	
Continue as planned at stage 1: 0.500 > *p* > 0.018			
Continue as planned at stage 2: 0.130 > *p* > 0.031			

The calculation was done with the R package MAMS

#### Randomization

The randomization will be done with https://www.randomizer.at/, a tool that is provided by the MED UNI Graz.

#### Data analysis

##### Calculation of the composite endpoint

In general, following the intention-to-treat (ITT) approach, all patients are included in the analysis. For scale variables, the means, standard deviations (SD), median values, and minimum/maximum values will be given. For ordinal variables, medians and minimum/maximum values will be calculated and reported. Frequencies and percentages will be given for nominal variables. Missing values are not considered when calculating the percentage.

The International Standards for the Neurological Classification of Spinal Cord Injuries (ISNCSCI) was chosen as the relevant clinical endpoint for this study. This score includes several subscores, which are listed in the table below.

**Table:** 

**Motor scores (MS)**	
“Upper Extremity Motor Score” (**UEMS**) UEMS = UEL + UER	“Lower Extremity Motor Score” (**LEMS**) LEMS = LEL + LER
“Upper Extremity Left” (UEL)	“Lower Extremity Left” (LEL)
“Upper Extremity Right” (UER)	“Lower Extremity Right” (LER)

**Table:** 

**Sensory scores (SS)**	
“Light Touch Total”(**LTT**)	“Pin Prick Total”(**PPT**)
LTT = LTL + LTR	PPT = PPL + PPR
“Light Touch Left” (LTL)	“Pin Prick Left” (PPL)
“Light Touch Right” (LTR)	“Pin Prick Right” (PPR)
**ISNCSCI score = UEMS + LEMS + LTT + PPT = TMSC** (**T**otal **M**otor and **S**ensory S**c**ore)	

If the study were to use each of these ISNSCI subscores separately as endpoints for hypothesis testing, alpha error correction would need to be applied because of multiple testing. However, this necessity implies a loss of power with the consequence that a larger sample would be needed to demonstrate the desired effect.

Due to the expected low incidence of spinal injuries that may ultimately be included in the study, it is already difficult to achieve the required sample size. For this reason, the ISNCSCI subscores are combined into a composite endpoint to avoid the necessity of an alpha error correction. With this approach, we follow the recommendations of the “U.S. Department of Health and Human Services Food and Drug Administration” (source: https://www.fda.gov/regulatory-information/search-fda-guidance-documents/multiple-endpoints-clinical-trials-guidance-industry).

However, to understand the treatment effect as best as possible independent of the hypothesis test, all subscores are exploratively analyzed and discussed.

For the final endpoint, however, the ISNCSCI score was not used, but an INSCSCI difference score that was determined from the baseline and follow-up measurements after 6 months. To avoid confusion, we will call this score “delta.TMSC.” The use of such a difference score simplifies the testing of the hypothesis and enables an uncomplicated description of the direction and type of the occurring change in the data [66, 67].

**Composite Endpoint: delta.TMSC = TMSC after 6 month - TMSC at baseline**

The entire measuring process is illustrated in the following figure. 
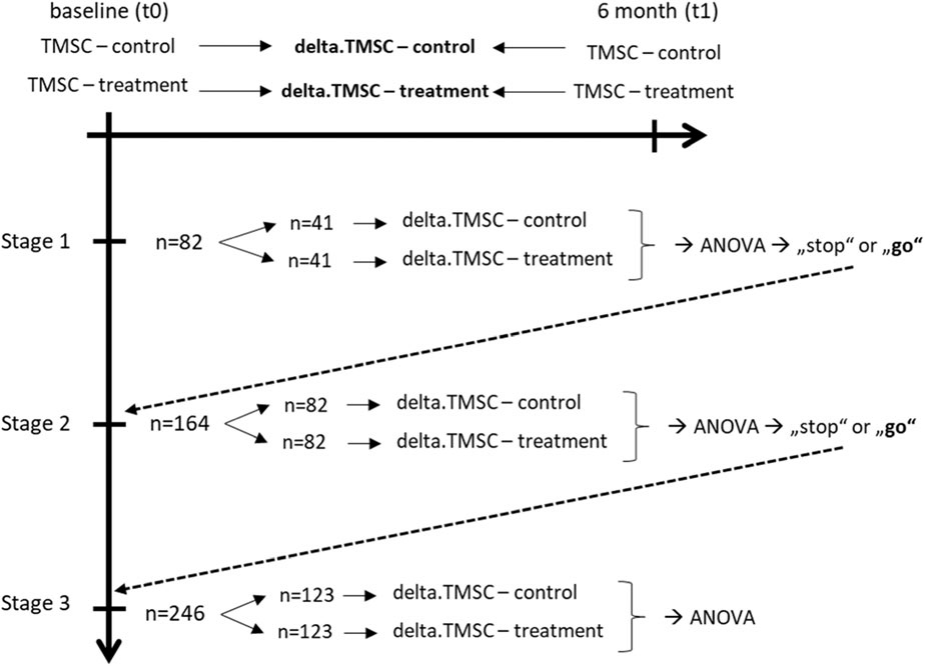


##### Hypothesis formulation and testing procedure

The aim of longitudinal assessments is to look for improvements over time. The treatment group is expected to achieve better results than the control group. To demonstrate the indicated treatment success, the delta.TMSCs is used.

Based on this score, the following hypotheses will be tested:

**H1: delta.TMSC treatment group > delta.TMSC control group**

**H0: delta.TMSC treatment group < = delta.TMSC control group**

Assuming that the data are normally distributed and that an ANOVA is robust against violations of the normal distribution, a randomized complete block design ANOVA is performed to compare the mean delta differences between the two groups. The three different neurological levels for stratification will be the block variable. Furthermore, the mean values of all subscores with their 95% confidence intervals are displayed per group. In addition, charts are created based on descriptive analysis to capture results at a glance.

Note: A problem with spinal trauma is that it usually occurs because of external force (e.g., car or motorcycle accident, fall from a great height) and is simultaneously associated with injuries to the extremities. Consequently, due to the chosen treatment strategy for the affected extremities (e.g., plaster, orthosis, surgical dressing), baseline measurements cannot be performed on them. To avoid bias in the study results as much as possible, these affected subscores will not be included in the calculation of the composite endpoint, even if the score is recorded at follow-up.

Sensitivity analysis (SA) is also performed to assess the robustness of the analyses. In particular, the effects of outliers and missing data will be the focus of this SA.

##### Secondary (exploratory) endpoints and control variables

Depending on the scales, the secondary endpoints will be reported descriptively as means with standard deviations, medians, and frequencies. If tests will be conducted, then an alpha less than 0.05 is considered as significant. All these tests are exploratory to get additional ideas on the impact of the treatment.

### Definition of collective analysis

All patients who fulfill the determined inclusion criteria will be used for the data analysis, independent of whether the intended shock wave therapy is performed as planned (intention-to-treat analysis).

### Handling of missing, unused, or faulty data; drop-outs; and terminations

In order to counteract missing values as best as possible, the study will be carried out according to the GCP principle (Good Clinical Practice). Additionally, a regular evaluation of the collected data will take place. If missing values are detected, an attempt will be made to subsequently determine them and/or to document the reasons.

Missing values concerning the primary endpoint are estimated according to the Last Observation Carry Forward principle (LOCF). If possible, the same procedure is used for the secondary endpoints. In all other cases, an analysis of the observed data will be carried out.

The systematically documented reasons for drop-outs will be evaluated to see if they can be used as a predictor for the endpoints and thereby additional insights into the therapy can be gained.

A sensitivity analysis will be performed in order to estimate the effect of missing data and outliers on the study endpoints.

## Supplementary material

A summary of all known adverse events and risks of the medical device and its properties is provided in the supplementary material. All measures for documentation and data recording, data management and storage, quality management including the regulations for monitoring and audits, reporting guidelines, and ethical and regulatory aspects are depicted in the supplement. Also, a detailed list of all study procedures can be found in the supplementary material.

## Discussion

Despite extensive efforts in the treatment of SCI, there is yet no causal, curative treatment approach available [10]. The current standard therapy in Austria is the early surgical decompression and stabilization [9, 11, 68] and in some cases the additional administration of steroids (methylprednisolone) in the acute phase of the injury (in the first 24–48 h, the latter being controversial) [10, 36–39].

ESWT is already being used in clinical practice such as chronic wounds, non-healing fractures, tendinopathies, myocardial ischemia, and lithotripsy. The application after acute spinal cord injury would thus be an expansion of indication for this therapy method, which has already been used in Austria for several years [12, 17, 19, 33, 34, 69].

ESWT could provide an easy-to-use, risk-free therapeutic method in the treatment of acute SCI in addition to surgical decompression with the aim of optimizing the clinical outcome.

The underlying study aims to investigate the effect of ESWT after acute traumatic spinal cord injury in humans within 48 h of trauma in order to intervene in the secondary injury phase with the objective to reduce the extent of neuronal damage.

## Trial status

Recruitment of study participants started in November 2020. Since then, recruitment takes place on an ongoing basis by admission of patients with acute spinal cord injuries. For patients who are included in the study, the follow-up phase after the baseline assessment will take approximately 6 months.

A planned interim analysis will be carried out after 41 patients have been included in each of both groups and all follow-up examinations have been completed. A further interim analysis will be planned after 82 patients per group. With unexpected major effects, the study will be prematurely terminated or, with low chances of success, will be canceled. An unplanned interim analysis will take place, should adverse events or side effects occur.

## Supplementary Information

**Additional file 1.** Flowchart / study visits

**Additional file 2.** Adverse Events

**Additional file 3.** Documentation

**Additional file 4.** Ethical and Regulatory Aspects

**Additional file 5.** Termination and patient exclusion

**Additional file 6.** Detailed study procedures

**Additional file 7.** Investigational Medical Device (IMD)

## Data Availability

Data can be made available upon reasonable request to the senior author after study closure.

## Abbreviations

AIS: American Spinal Injury Association (ASIA) Impairment Scale
AUVA: Allgemeine Unfallversicherungsanstalt
CCI: Charlson Comorbidity Index
CT: Computed tomography
DSMB: Data Safety Monitoring Board (Datensicherheitskomitee)
eCRF: Electronic case report form
ESWT: Extracorporeal shockwave therapy (extrakorporale Stoßwellentherapie)
GCP: Good Clinical Practice
GCS: Glasgow Coma Scale
GRT: Grasp and Release Test
ISNCSCI: International Standards for Neurological Classification of Spinal Cord Injury
ISS: Injury Severity Score
LEMS: Lower Extremity Motor Score
LEL: Lower extremity left
LER: Lower extremity right
LTL: Light touch left
LTR: Light touch right
LOCF: Last Observation Carry Forward principle
MAS: Modified Ashworth Scale
MF: Motor function
MPG: Medical device law (Medizinproduktegesetz)
na: Not applicable
NCI: National Cancer Institute
nd: Not done
NHPT: Nine-Hole Peg Test
NRS: Numeric Rating Scale
PSFS: Penn Spasm Frequency Scale
PI: Principal investigator
PPL: Pin prick left
PPR: Pin prick right
SAP: Statistical Analysis Plan
SCI: Spinal cord injury
SCIM: Spinal cord independence measure
SF: Sensory function
SPSS: Statistical Package for Social Sciences
TMSC: Total Motor and Sensory Score
TUG: Timed Up and Go Test
UEMS: Upper Extremity Motor Score
UEL: Upper extremity left
UER: Upper extremity right
WISCI II: Walking Index for Spinal Cord Injury II
