# Supplementary material for: The effect of extracorporeal shock wave therapy in acute traumatic spinal cord injury on motor and sensory function within 6 months post-injury: a study protocol for a two-arm three-stage adaptive, prospective, multi-center, randomized, blinded, placebo-controlled clinical trial
Source: Trials. 2022 Apr 1;23:245. doi: 10.1186/s13063-022-06161-8 (PMC8973563; doi:10.1186/s13063-022-06161-8)
Supplement: Supplementary file 1 — Additional file 1. Flowchart / study visits [file 13063_2022_6161_MOESM1_ESM.docx]

Flowchart


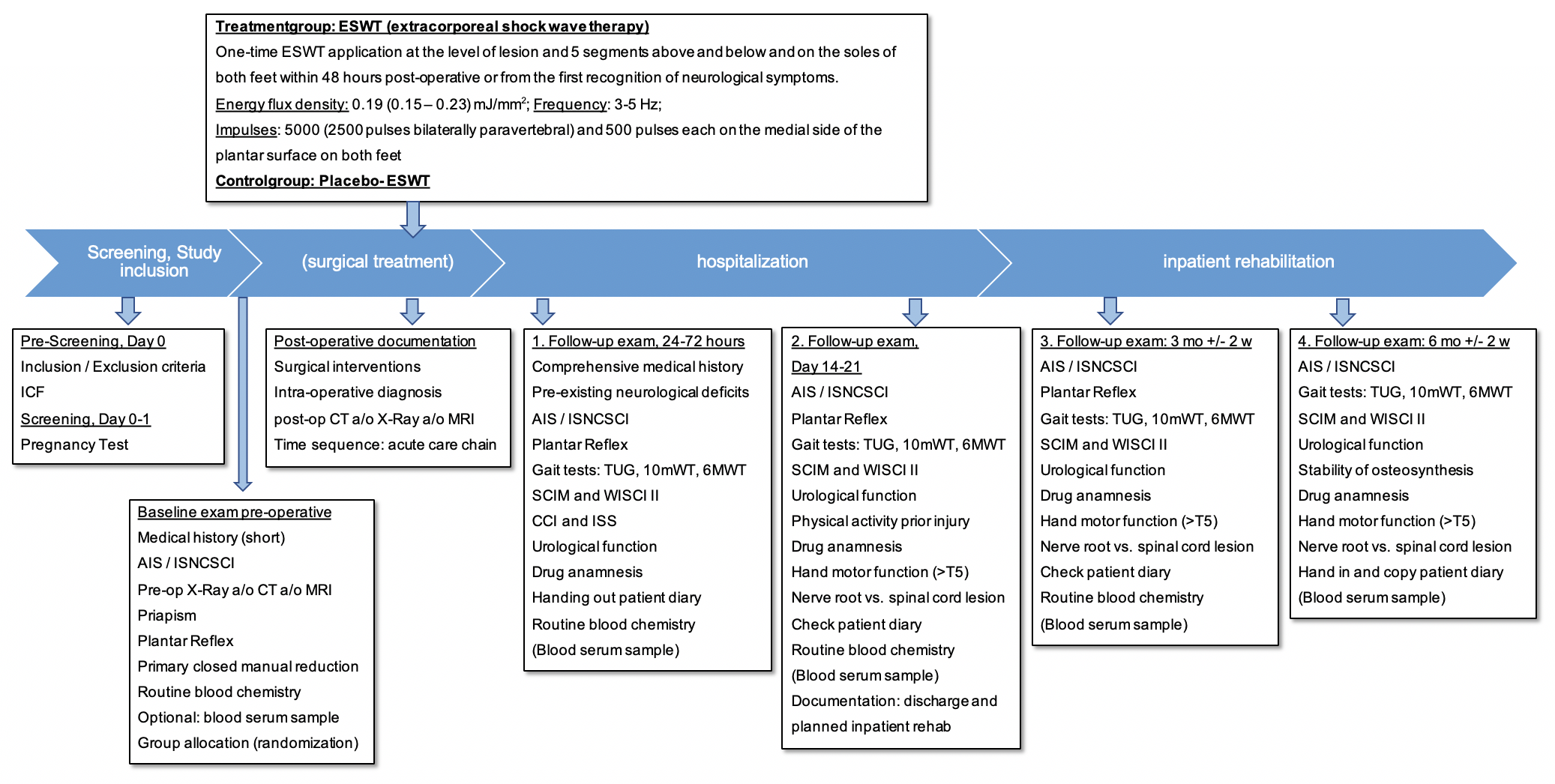


| Visits | Visit 1 | Visit 2 | Visit 3 | Visit 4 | Visit 5 | Visit 6 | Visit 7 | Visit 8 |
| --- | --- | --- | --- | --- | --- | --- | --- | --- |
|  | Pre-Screening | Screening | Baseline  pre-operative | Post-operative documenta-tion | Follow-up 1 | Follow-up 2 | Follow-up 3 | Follow-up 4 |
|  |  |  |  |  | 24-72 hours | Day 14-21 | 3 Mon  +/-2 We | 6 Mon  +/-2 We |
| Informed Consent NeuroWave | x |  |  |  |  |  |  |  |
| Informed Consent ASCIS (only in Austria) | x |  |  |  |  |  |  |  |
| Inclusion/Exclusion Criteria | x |  |  |  |  |  |  |  |
| Pregnancy test (women with child bearing potential) |  | x |  |  |  |  |  |  |
| Short Medical History |  |  | x |  |  |  |  |  |
| AIS/ISNCSCI |  |  | x |  | x | x | x | x |
| Pre-op CT/MRI/X-ray |  |  | x |  |  |  |  |  |
| Priapism (in male patients) |  |  | x |  |  |  |  |  |
| Plantar Reflex (Babinski) |  |  | x |  | x | x | x |  |
| Primary closed manual reduction |  |  | x |  |  |  |  |  |
| Documentation of routine blood chemistry |  |  | x |  | x | x | x |  |
| Optional: blood serum samples |  |  | (x) |  | (x) | (x) | (x) | (x) |
| Group allocation: Randomization |  |  | x |  |  |  |  |  |
| Surgical interventions and intra-operative diagnosis |  |  |  | x |  |  |  |  |
| Post-op CT/MRI/X-ray |  |  |  | x |  |  |  |  |
| Time course of acute care chain |  |  |  | x |  |  |  |  |
| Documentation of ESWT |  |  |  | x |  |  |  |  |
| Comprehensive Medical History |  |  |  |  | x |  |  |  |
| Pre-existing neurological deficits |  |  |  |  | x |  |  |  |
| Gait tests |  |  |  |  | x | x | x | x |
| SCIM an d WISCI II |  |  |  |  | x | x | x | x |
| CCI and ISS |  |  |  |  | x |  |  |  |
| Urological function |  |  |  |  | x | x | x | x |
| Drug anamnesis / concomitant Medication |  |  |  |  | x | x | x | x |
| Patient`s diary |  |  |  |  | x | x | x | x |
| Retrospective: physical activity prior trauma |  |  |  |  |  | x |  |  |
| Hand motor function (>T5) |  |  |  |  |  | x | x | x |
| Nerve root vs. spinal cord lesion |  |  |  |  |  | x | x | x |
| Documentation of discharge and planned inpatient rehabilitation |  |  |  |  |  | x |  |  |
| AEs und SAEs |  |  |  |  | x | x |  |  |
| CT/MRI/X-ray: Stability of osteosynthesis |  |  |  |  |  |  |  | x |
