# Supplementary material for: The effect of extracorporeal shock wave therapy in acute traumatic spinal cord injury on motor and sensory function within 6 months post-injury: a study protocol for a two-arm three-stage adaptive, prospective, multi-center, randomized, blinded, placebo-controlled clinical trial
Source: Trials. 2022 Apr 1;23:245. doi: 10.1186/s13063-022-06161-8 (PMC8973563; doi:10.1186/s13063-022-06161-8)
Supplement: Supplementary file 2 — Additional file 2. Adverse Events [file 13063_2022_6161_MOESM2_ESM.docx]

**Adverse Events**

**Summary of known and potential Risks of the Medical Device**

Provide a summary of all known adverse events and risks of the medical device and other interventions (preferably in a table). Define device deficiencies, use the instructions for use (IFU)

In addition to adverse event reporting, non-medical complaints such as malfunction, misuse, use error not leading to an adverse event, shall be documented throughout the clinical investigation and shall be reported as specified by local (or national?) law.

All complaints of a non-medical nature shall be handled under the sponsor's quality management system.

**Adverse Event (AE)**

A clinical trial adverse event is any untoward medical occurrence, unintended disease or injury, or untoward clinical signs (including abnormal laboratory findings) in a patient or clinical trial subject C or not related to the investigational medical device. This includes events related or not to a comparator and procedures involved.

Surgical procedures themselves are not AEs; they are therapeutic measures for conditions that require surgery. The condition for which the surgery is required may be an AE. Planned surgical measures permitted by the clinical trial protocol and the condition(s) leading to these measures are not AEs, if the condition leading to the measure was present prior to inclusion into the trial.

**Serious Adverse Event (SAE)**

If a Serious adverse event (SAE) occurs, the Investigator must alert the sponsor without unjustified delay to any AE (whether causally or not) from this study that results in one of the following outcomes, or is significant for any other reason:

- death (excluding death from progressive disease)
- a life-threatening experience – refers to an event in which the subject was at risk of death at the time of the event; it does not refer to an event which hypothetically might have caused death if it were more severe
- initial hospitalization, or prolongation of existing inpatients hospitalization
- persistent or significant disability or incapacity
- congenital anomaly or birth defect
- occur a malignant tumor

Medical judgment should be exercised in deciding whether an AE/ADE is serious in other situations. Important AE/ADEs that are not immediately life-threatening or do not result in death or hospitalization but may jeopardize the subject or may require intervention to prevent one of the other outcomes listed in the definition above, should also be considered serious.

Patients should be closely followed for adverse events while receiving treatment with the investigational device and for 3 weeks after discontinuation from study therapy in order to detect delayed toxicity.

Events not considered to be serious adverse events are hospitalizations for the:

- routine treatment or monitoring of the studied indication, not associated with any deterioration in condition
- treatment, which was elective or pre-planned, for a pre-existing condition that did not worsen
- treatment on emergency, outpatient basis for an event not fulfilling any of the definitions of serious given above and not resulting in hospital admission.

Disease progression will not be reported as AE or SAE unless the progression is unexpected in severity or early occurrence. Progressive disease will be appropriately documented on the case report form as part of the efficacy parameters.

**Adverse Device Effect (ADE)**

An Adverse device effect (ADE) is an adverse event, which is related to the use of an investigational medical device. All untoward and unintended responses to an IMD related to any dose administered. This definition includes adverse events resulting from insufficient or inadequate instructions for use, deployment, implantation, or operation, or any malfunction, use error or intentional misuse of the investigational medical device.

**Serious Adverse Device Effect (SADE), Anticipated Serious Adverse Device Effect (ASADE) and Unanticipated Serious Adverse Device Effect (USADE)**

Serious adverse events that are considered to be related to the medical device are serious adverse device effects (SADE). SADE’s comprise unanticipated serious adverse device effects (USADE) and anticipated serious adverse device effects (ASADE).

USADE means any serious adverse device effect which by its nature, incidence, severity or outcome has not been identified in the current version of the risk analysis report.

ASADE means any serious adverse device effect which by its nature, incidence, severity or outcome has been identified in the current version of the risk analysis report.

**Definition of Device Deficiency**

Please find the definition of device deficiencies in the attached document: MTS_RA_OW100_Device-Deficiency_E_A.pdf

**Pregnancy**

Any pregnancy that occurs during study participation must be reported to the investigator/sponsor immediately.

The pregnancy must be followed up to determine outcome (including premature termination) and status of mother and child. Pregnancy complications (including spontaneous abortions) and elective terminations must be reported as an AE or SAE.

Any SAE occurring in association with a pregnancy brought to the investigator’s attention after the subject has completed the study and considered by the investigator as possibly related to the IMD, must be promptly reported to the sponsor.

As the consent for the study does not cover the consent for the follow-up of the pregnancy, a separate consent has to be obtained.

**Severity of Adverse Events (AE)**

The severity of clinical AEs/ADEs is graded on a three-point scale: mild, moderate, severe, and reported on specific AE pages of the CRF.

If the severity of an AE/ADE worsens during medical device administration, only the worst intensity should be reported on the AE page. If the AE lessens in intensity, no change in the severity is required.

**Mild**Event may be noticeable to subject; does not influence daily activities; the AE/ADE resolves spontaneously or may require minimal therapeutic intervention.

**Moderate**Event may make subject uncomfortable; performance of daily activities may be influenced; intervention may be needed; the AE/ADE produces no sequelae.

**Severe**Event may cause noticeable discomfort; usually interferes with daily activities; subject may not be able to continue in the study; the AE/ADE produces sequelae, which require prolonged therapeutic intervention.

A mild, moderate or severe AE/ADE may or may not be serious. These terms are used to describe the intensity of a specific event (as in mild, moderate, or severe myocardial infarction). However, a severe event may be of relatively minor medical significance (such as severe headache) and is not necessarily serious. For example, nausea lasting several hours may be rated as severe, but may not be clinically serious. Fever of 39°C that is not considered severe may become serious if it prolongs hospital discharge by a day. Seriousness rather than severity serves as a guide for defining regulatory reporting obligations.

**Causality**

For all, the investigator will assess the causal relationship between the medical device and the AE using his/her clinical expertise and judgment according to the following algorithm that best fits the circumstances of the AE:

Unrelated

- May or may not follow a reasonable temporal sequence from administration of the study product
- Is biologically implausible and does not follow known response pattern to the suspect medical device (if response pattern is previously known)
- Can be explained by the known characteristics of the subject’s clinical state or other modes of therapy administered to the subject.

Unlikely

- There is a reasonable temporal relation between the AE and the medical device, but there is a plausible other explanation for the occurrence of the AE.

Possibly

Follow a reasonable temporal sequence from administration of the medical device.

- The AE may equally be explained by the study subject’s clinically state, environmental or toxic factors, or concomitant therapy administered to the study subject.
- The relationship between the medical device and AE may also be clinically plausible.

Probably

- Follows a reasonable temporal sequence from administration of the medical device, and plausible reasons point to a causal relation with the medical device.

Related

- Follows a reasonable temporal sequence form administration of the medical device.
- Follows a known response pattern to the medical device (if response pattern is previously known).
- No other reasonable cause is present.

Not assessable

- The causal relationship between the medical device and the AE cannot be judged.

**Reporting Procedures**

**Reporting Procedures for Adverse Events (AEs)**

A special section is designated to adverse events in the case report form. The following details must thereby be entered:

- Type of adverse event
- Start (date and time)
- End (date and time)
- Severity (mild, moderate, severe)
- Serious (no / yes)
- Unexpected (no / yes)
- Outcome (resolved, resolving, not resolved, resolved with sequelae, unknown, fatal)
- Relation to medical device (Related/ Probably/ Possibly/ Unlikely/ Not related/ Not assessable)

Adverse events are to be documented in the case report form in accordance with the above mentioned criteria.

**Reporting Procedures for Serious Adverse Events (SAEs)**

In the event of serious, the investigator has to use all supportive measures for best patient treatment. A written report is also to be prepared and made available to the clinical investigator immediately. The following details should be at least available:

- Patient number
- Patient: initials, age in years, sex
- The suspected medical device
- The adverse event assessed as serious
- Short description of the event and outcome
- Device related or non-device related

The written report is divided into two parts:

- Initial report: Informs about what has happened (AE/ADE assessed as serious), if there is a relationship to the medical device, and which action was set.
- Follow up-Report: informs about the outcome

The Sponsor is responsible for the classification of adverse events and ongoing safety evaluation of the clinical investigation and shall:

- review the investigators assessment of all adverse events and determine and document in writing their seriousness and relationship to the investigational device
- review all devices deficiencies and determine and document in writing whether they could have led to a serious adverse device effect
- report or ensure the reporting of all SAEs, whether or not related to the medical device, to the EC and regulatory authorities (BASG)
