# Supplementary material for: The effect of extracorporeal shock wave therapy in acute traumatic spinal cord injury on motor and sensory function within 6 months post-injury: a study protocol for a two-arm three-stage adaptive, prospective, multi-center, randomized, blinded, placebo-controlled clinical trial
Source: Trials. 2022 Apr 1;23:245. doi: 10.1186/s13063-022-06161-8 (PMC8973563; doi:10.1186/s13063-022-06161-8)
Supplement: Supplementary file 3 — Additional file 3. Documentation [file 13063_2022_6161_MOESM3_ESM.docx]

**Documentation**

The accomplishments of the study in agreement with the EN ISO 14155 guidelines, the MD and the clinical investigational plan as well as the trueness of all data documented in the CRF are the responsibility of the Investigator. All collected data of this study have to be recorded in the CRF by appropriate authorized persons. This is also valid for data of patients, who dropped-out of the study.

The Investigator records the participation in a special identification list of patients. This list gives the possibility to a later identification of the patients and contains the patient number, full name, date of birth and the date of the enrollment in the study. The identification list of patients remains in the study center after the closure of the study. Additionally, the participation of the patient in this clinical study has to be recorded in the patient chart (investigational device, number of patient or randomization, start and end of the study).

Further it has to be assured, that this person, who is responsible for the documentation in the eCRF, can be identified. A list with signatures and identification code of the persons, who are allowed to make entries in the eCRF, will be archived in the Investigator Site File and the Trial Master File.

**Data Recording (eCRF)**

It is the responsibility of the investigator to document all data of the clinical study correctly and completely into the database. Corrections in the eCRF (electronic Case Report Form) can only be made by people authorized by the principal investigator and have to be justified. Corrections are filed in a way that previous data can be recalled. All data and corrections are traced with date, time and person.

**Trial Folders**

The trial folders should contain the complete documentation of the trial. Individually or together, they should allow the evaluation of the trial conduct and its data quality.

**Trial Master File (TMF)**

The paper-based TMF, established at the beginning of the trial and secured in a safe place, contains all essential documents that demonstrate that the trial is conducted in accordance with regulatory requirements and ICH GCP. All documents will be maintained and updated as appropriate throughout the trial. Previous versions of the documents must be retained in the TMF and will be clearly labelled as outdated or will be relocated in a section for outdated documents. The TMF is archived at the end of the study for 15 years.

**Investigator Site File (ISF)**

The paper-based ISF, established at the beginning of the trial will be secured in a safe place (the file is provided to the site at the site initiation visit). It contains all essential documents maintained by the PI(s). All documents will be maintained and updated as appropriate throughout the trial. Previous versions of the documents must be retained in the ISF and will be clearly labelled as outdated or will be relocated in a section for outdated documents. Within the Monitoring, the ISF will be checked up on actuality and completeness in accordance with the formalities. After completion or discontinuation of the study this ISF has to be kept for 15 years.

**Data Storage**

**Storage duties of the Sponsor**

The Sponsor has to keep all essential documents of the clinical investigation after completion or discontinuation of the study for a minimum of 15 years. The Sponsor has to archive all study-relevant documents in accordance with the legal regulation.

**Storage duties of the Investigator**

The Investigators have to keep all records and documents, which are related with the study or the allocation of investigational device (e.g. data entry form, informed consent form, list of the allocations of investigational devices and further relevant documents), for a minimum of xx years.

Medical records and other original data have to be kept for the longest possible duration, which the hospital, the institution or the private praxis permits.

**Access to the investigational device and Documentation**

Access to the investigational device shall be controlled. Medical device must only be used in the clinical trial in accordance with the clinical trial protocol.

The sponsor has to keep records, to which document the current location of all delivered investigational medical devices including their return or disposal.

The investigator or an authorized representative has to keep records, to document the receipt, use, return and disposal of the care IMD. These records should include the following points:

1. Date of receipt
2. Identification of the IMD (batch number, serial number or an unique code)
3. Expiry date (if applicable)
4. Date of application
5. Identification of test subject
6. date of return/explanation from test subject (if applicable)
7. Date of return of unused, expired or non-functional IMDs (if applicable)
8. Training of the user

**Data Management**

Data are managed and collected by the Askimed (https://www.askimed.com) software. Askimed is a cloud-based web platform that provides an all-in-one solution for medical studies of any size. Askimed main features are (1) data collection based on electronic case report forms (eCRF), (2) data management including a permission system for study collaborators and (3) data preparation for data analysis. Each study collaborator uses Askimed as the central information source and is guided by the platform in all three study phases. Askimed integrates user friendly tools for error elimination, data reproducibility and data security. All study data is stored in a MySQL database, and the software uses state-of-the-art technologies to secure all server communication and user actions. Its internal architecture is designed to manage and analyze millions of data items from eCRFs. For eCRF creation itself, Askimed provides a graphical tool (Askimed Editor) to generate the questionnaire dynamically. Several different questionnaire types (e.g. textboxes, comboboxes, radio buttons, pictures, numbers) are available and plausibilities as well as jumps can be coded by using Askimeds eCRF engine. To support different languages, the eCRF logic is defined once and can be used for all available study languages. Changes are tracked by the Askimed system and allows to update the CRF at any time. Data collection can be done via a web browser or by using a Desktop client, in which data is synchronized with the Askimed database. Askimed also includes an audit trail, study monitoring (e.g. query workflow, interview signing), management of collaborators and probands as well as the creation of data dictionaries of all available variables.
The Askimed system has been developed at the Medical University of Innsbruck and is used in several major epidemiological studies and patient registers. Overall, Askimed provides an efficient way to store all study specific data in one central repository and improves the overall quality compared to paper-based CRFs.

Validation of data is made by programmed checks of range, validity and consistency. If necessary, queries are made by the study software or an authorized person. Based on the queries the investigator can check and clarify discrepancies.

For completion of the study after the record of all entries and clarification of all queries, the data base will be closed. This process has to be documented.

**Protocol Deviations**

1. Statement specifying that the investigator is not allowed to deviate from the CIP, except as specified in EN ISO 14155 4.5.4 b:

- Requests for deviations, and reports of deviations, if the deviation affects subject's rights, safety and wellbeing, or the scientific integrity of the clinical investigation;
- Under emergency circumstances, deviations from the CIP to protect the rights, safety and well-being of human subjects may proceed without prior approval of the sponsor and the EC. Such deviations shall be documented and reported to the sponsor and the EC as soon as possible.

1. Procedures for recording, reporting and analyzing of the CIP deviations.
2. Notification requirements and time frames.
3. Corrective and preventive measures and criteria for the exclusion of the principal investigator.

**Quality Management**

Training, monitoring and audits are performed for quality assurance within this clinical study. Monitoring and auditing procedures developed or endorsed by the Sponsor will be conducted, in order to comply with ICH-GCP guidelines (EN ISO 14135) and local legal requirements to ensure acceptability of the study data.

**Qualifications**

The Sponsor is responsible for selecting the Investigator(s)/Institution(s). Each Investigator should be qualified by training and experience and should have adequate resources. Each individual involved in conducting a trial should be qualified by education, training and experience to perform his or her respective task(s) (see ICH GCP E6).

**Monitoring**

The Investigator must grant direct access to on-site study documentation, including the patient’s notes, to allow audits or inspections to be performed. No action will be taken that might infringe the patients’ confidentiality.

Monitoring visits by representatives of the Sponsor will be carried out to review study plan compliance, to compare CRFs and individual patient’s medical records, to perform accounting of study material, and to ensure that the study is being conducted according to pertinent regulatory requirements. CRF entries will be verified with source documentation. The frequency and duration of Monitoring visits will be determined according to clinical site accrual, site performance, adherence to the protocol, and data quality.

Further the Monitor confirms that:

- the clinical investigational plan is fulfilled and deviations have to be discussed with the investigator, documented and reported to the sponsor
- the product is used complied to the clinical investigational plan and changes of the product, its application or with the clinical investigational plan have to be reported to the sponsor
- for the investigator a sufficient amount of test subjects and products are provided
- a signed and dated consent form exists for each test subject at the time of admission and before initiation
- Inspection of the Investigator Site File (ISF)
- Documentation of the patient status
- CRF-data verification with the source data
- Evaluation of the SAEs reports according to the regulations
- a proceed regarding balancing and traceability of the products exists
- service and calibration of the devices are carried out and documented
- resignation of a test subject and/or non-compliance of the rules must be documented, discussed with the investigator and reported to the sponsor

The monitor has to treat all information confidential and protect the privacy of the patients.

**Audits and Inspections**

Regulatory authorities, the ethics committees, and Sponsor’s delegates may perform on-site inspections or audits, for which the Investigator must provide support at all times.

During an audit following issues among other things will be inspected:

- Performance of the clinical trial according to the investigation plan
- Data validity
- Quality of the clinical trial according to the EN ISO 14155 guidelines

After each external audit an audit-certificate by the auditor has to be sent to the Investigator. This certificate has to be kept in the Investigator Site File (ISF) to evidence the audit to the regulatory authorities in the case of an inspection by them. The audit-report is sent to the Sponsor of the study. An audit-certificate will be attached to the final report at the end of the study. Additionally, according to the Austrian Medical Device Law (MPG) audits and inspections by regulatory authorities may be performed.

**Reporting**

For the documentation of the progress and development of the study, protocols about the meetings of the various group committees are necessary.

**Final Study Report**

All information regarding this clinical study has to be kept confidential. The statistical analysis and the integrated final study report will be prepared according EN ISO 14155 and finalized within 12 months after last patient last visit (LPLV) took place. The final study report will be reviewed and signed by the Sponsor, the Coordinating Investigator and all further responsible persons. All information in that report is strictly confidential.

Please choose:

- The coordinating investigator will sign the final study report of the clinical trial. This confirms that the report describes implementation and results of the clinical trial by the best of his knowledge.
- The investigator with most patients becomes the coordinating investigator of the final study report. If the coordinating investigator is unable to meet his obligation, the sponsor will determine a new coordinating investigator of the final study report.
- The coordinating investigator, employed by the sponsor, signs the final study report of this clinical trial. This confirms that the report describes implementation and results of the clinical trial by the best of his knowledge.

**Publications**

Data of the study will be published according to the publication guidelines. Publication of the study results is aspired in an adequately high ranked journal as soon as data analysis is finished.

The five best recruiting sites are entitled to co-authorship. The remaining persons will be entitled as “Group authorship” NeuroWAVE study group. This means that each person will be entered as collaborator names in Medline citation.

The sequence of authors will arise from the number of included subjects.

**Amendments**

After the protocol has been submitted to an ethics committee (EC), any substantial change will require a formal amendment. The amendment must be signed by all of the signatories to the original protocol. Once the study has started, amendments should be made only in exceptional cases. The ethics committees must be informed of all amendments. If necessary, approval must be sought for ethical aspects and must also be obtained from the competent authorities.
