# Supplementary material for: The effect of extracorporeal shock wave therapy in acute traumatic spinal cord injury on motor and sensory function within 6 months post-injury: a study protocol for a two-arm three-stage adaptive, prospective, multi-center, randomized, blinded, placebo-controlled clinical trial
Source: Trials. 2022 Apr 1;23:245. doi: 10.1186/s13063-022-06161-8 (PMC8973563; doi:10.1186/s13063-022-06161-8)
Supplement: Supplementary file 4 — Additional file 4. Ethical and Regulatory Aspects [file 13063_2022_6161_MOESM4_ESM.docx]

**Ethical and Regulatory Aspects**

**Responsibilities of Sponsor and Investigator**

The sponsor of this clinical trial will assume responsibility for inducement, organization and financing of the implementing trial according to the EN ISO 14155. The procedures set out in this study protocol are designed to ensure that the Sponsor and the Investigator comply with the principles of ICH-GCP the Declaration of Helsinki and the EN ISO 14155 guideline concerning the conduct, evaluation and documentation of the study. The study will also be performed adhering the local legal conditions and requirements. Each Investigator has to confirm this by signing the study protocol.

Responsibilities of the sponsor:

- Verification of the understanding of the instructions for use (IFU)
- Verification of the understanding of treatment schedule
- Ensuring for enough time and capacity for the implementation of this study
- Correct collection and documentation of data, reporting
- Provision of all data to the sponsor, monitor or relevant authorities for audits or inspections
- Assurance for the confidential handling of patients data and information

The Principal Investigator accepts the responsibility for the conduct of this clinical trial at this study site according to the EN ISO 14155.

**Approval of Ethics Committee and Notification to the Authority**

Prior to study start, the study protocol and/or other appropriate documents will be approved by the appropriate ethics committees and competent authorities.

**Patient Information and Consent Form**

Every patient has to give his/her written consent before the participation in the clinical trial. Before the patient gives his/her written consent the patient has to be informed completely in oral and written form in an understandable manner about character, importance, relevance and consequences of the clinical trial.

The content of the consent information is documented on the Patient information and Informed Consent form. The patient will be notified, if essential findings about the MD appear during the study.

The Informed Consent of the patient about the participation in the clinical trial has to be dated and signed by the patient and the Investigator. The patient receives a copy of the signed and dated Patient Information and Informed Consent Form. The Investigator stores the original signed and dated exemplar in the Investigator Site File.

It has to be explicitly pointed out, that before patient sign the Informed Consent form it is not allowed to perform any study specific actions with the patient.

The investigator, his / her representative, must inform the patient that there is no obligation to participate in the clinical trial and the option to leave the clinical trial at any time without giving reasons. If the patient has received and understood all information, there is sufficient time for questions and the patient agrees to participate, the patient should date and sign the declaration of consent.

No activities relevant to the clinical trial will be undertaken before the consent of the patient has been obtained.

If new safety analyzes lead to a significant change in the risk analysis and risk assessment of the test product, the declaration of consent must be reviewed and, if necessary, revised.

**Consent for adult patients who are physically incapable of sign the consent**

For patients who are physically incapable to sign the consent to participate by their selves, but are awake, oriented and willing to participate, the investigator and one additional witness confirm the verbal consent with their signature.

**Blinding and Unblinding**

**Blinding**

The study will be blinded for treatment so that the patient and the assessor of the neurological assessment and gait analyses are unaware of the treatment group assigned. The randomization list will be opened after the study database has been locked. The patients randomized to the non-treatment group, who will receive placebo treatment after surgery, will receive a shockwave application with a dummy sonic head, the connected device is the same as in treatment group.

**Emergency Envelopes**

As the shockwave treatment has no influence on the emergency treatment, we waive the use of emergency envelopes. If the information is needed, the monitor can be asked. The reason, time and date of un-blinding has to be documented.

**Unblinding ahead of schedule**

If Data Safety Monitoring Board (DSMB) decides that unblinding ahead of schedule should be performed.

**Scheduled Unblinding**

The regular unblinding will be done after the completion of data entry (after closure of data base).

**Insurance**

The Sponsor will take out reasonable third-party liability insurance cover in accordance with local legal requirements. The civil liability of the Investigator, all persons instructed and the hospital, practice or institute in which they are employed and the liability of the Sponsor in respect of financial loss due to personal injury and other damage that may arise as a result of the carrying out of this study are governed by the applicable local law.

As a precautionary measure, the Investigator, the persons instructed and the hospital, practice or institute are included in such cover in terms of their work done in carrying out the study to the extent that the claims are not covered by their own professional indemnity insurance.

The Sponsor will arrange for patients participating in this study to be insured against financial loss due to personal injury caused by the study medication being tested or by medical steps taken in the course of the study. Such insurance is taken out by the Sponsor in accordance with or by way of analogy to both the Austrian and the other participating countries drug law.

**Data Protection and Confidentiality**

All local legal requirements regarding data protection will be adhered to. All study findings and documents will be regarded as confidential. The Investigator and members of the research team must not disclose any information without prior written approval from the Sponsor.

The pseudonymity of patients participating must be maintained. Throughout documentation and evaluation, the patients will be identified on CRFs and other documents by xxx. Documents that identify the patient personally (e.g., the signed informed consent, patient identification list) must be maintained in confidence by the Investigator. The patients will be informed in the ICF that all study findings will be stored on computer and handled in strictest confidence.

**Financing**

The present clinical trial is funded by AUVA (Austrian Worker`s Compensation Board).

**Regulatory Aspects**

The processes set out in this study protocol are designed to ensure that the Sponsor and the Investigator abide the principles of the EN ISO 14155 and the Declaration of Helsinki concerning the conduct, evaluation and documentation of the study. The study will also be performed in compliance with the local legal conditions and requirements. Each Investigator has to confirm this by signing the study protocol.

**Data Safety and Monitoring Board**

The Data Safety and Monitoring Board is charged with overseeing the safety of the patients and reviewing the results according to the DSMB charta. The members are:

Mag. Dr. Elisabeth Ponocny-Seliger

Coaching, empirische Sozialforschung & Gender Research

Spaungasse 19/2/8

A-1200 Wien

Tel.: +43 676 5991641‬

e-mail: office@gender-research.at

Prof. Dr. med. Armin Curt
Zentrum für Paraplegie
Universitätsklinik Balgrist
Forchstrasse 340
CH 8008 Zürich

Tel.: +41 44 386 11 11

e-mail: [Armin.Curt@balgrist.ch](mailto:Armin.Curt@balgrist.ch)

Priv.-Doz. Dr. med. Stefan Leis, MME
Leitender Oberarzt Neurologie
Stabsstelle Medizindidaktik der PMU
Uniklinikum Salzburg, Christian-Doppler-Klinik
Universitätsklinik für Neurologie der PMU 
Ignaz-Harrer-Str. 79

A-5020 Salzburg
Tel.: +43 (0)5 7255-0
e-mail: [s.leis@salk.at](mailto:s.leis@salk.at)

For further information, please see the Guideline on Data Monitoring Committees (Doc. Ref. EMEA/CHMP/EWP/5872/03 Corr).
