# Supplementary material for: The effect of extracorporeal shock wave therapy in acute traumatic spinal cord injury on motor and sensory function within 6 months post-injury: a study protocol for a two-arm three-stage adaptive, prospective, multi-center, randomized, blinded, placebo-controlled clinical trial
Source: Trials. 2022 Apr 1;23:245. doi: 10.1186/s13063-022-06161-8 (PMC8973563; doi:10.1186/s13063-022-06161-8)
Supplement: Supplementary file 5 — Additional file 5. Termination and patient exclusion [file 13063_2022_6161_MOESM5_ESM.docx]

**Termination and patient exclusion**

**Premature termination of the clinical trial**

The clinical trial is terminated prematurely if:

- the patient recruitment rate is inadequate to reach the aims of the study
- serious, unexplained problems with the quality of the collected data occur
- unpredictable circumstances have occurred in the respective test center which do not allow continuation of the clinical trial
- the early detection of superiority or inferiority of the treatment group (as defined by interim analysis) is provided
- unacceptable risks and toxicities have occurred (decision after new risk analysis and evaluation of the test product)
- new scientific information comes up during implementation of the clinical trial which does not allow the continuation of the clinical trial for ethical or scientific reasons

**Reasons for patient exclusion after enrollment (drop-out)**

Patients have the option to leave the clinical trial at any time without giving reasons.

Additional neurological diseases or diseases that can cause damage to the nervous system and that are diagnosed or communicated after enrollment and the respective patient was therefore included in the study by error, lead to exclusion from the study.

**Pre-existing deficits leading to the subsequent exclusion of study:**

- Pre-existing functionally severe psychiatric disorders
- Neurological pre-existing diseases that affect motor and sensory function and therefore might reduce or mask the effect of the study intervention or impede the study related examinations:
  - Non-traumatic paresis or plegia
  - Peripheral nerve lesions above the level of the lesion
  - Radicular lesions
  - Pre- existing myelopathy with neurological symptoms which were already present prior to the acute spinal cord injury
  - Demyelinating diseases
