# Supplementary material for: The effect of extracorporeal shock wave therapy in acute traumatic spinal cord injury on motor and sensory function within 6 months post-injury: a study protocol for a two-arm three-stage adaptive, prospective, multi-center, randomized, blinded, placebo-controlled clinical trial
Source: Trials. 2022 Apr 1;23:245. doi: 10.1186/s13063-022-06161-8 (PMC8973563; doi:10.1186/s13063-022-06161-8)
Supplement: Supplementary file 6 — Additional file 6. Detailed study procedures [file 13063_2022_6161_MOESM6_ESM.docx]

**Detailed study procedures**

**Pre-Screening**

Check fulfillment of inclusion and exclusion criteria

Written informed consent

**Screening**

Pregnancy test for women with child bearing potential

**Baseline examination pre-operative**

Short medical history

AIS/ISNCSCI

Diagnosis SCI according to radiological imaging via CT and/or X-Ray and/or MRI:

Level of fracture

AOSpine fracture classification

Cause of injury to the spinal cord (if no fracture is present): free text or “not specified”

Posterior vertebral wall affected? yes/no

Mechanism of injury (penetrating, not penetrating, not specified)

Priapism (for male patients only): yes/no

Plantar Reflex

Primary closed manual reduction prior to surgical intervention: yes/no

Documentation of parameters from routine blood chemistry

OPTIONAL: Baseline blood sample at admission and prior to the surgical intervention and the application of the ESWT: one serum tube (8ml)

Block randomization (Group allocation)

**Operative treatment**

**Study intervention**

Opening of randomization envelope

ESWT: Begin, number of impulses, energy flux density, frequency, end, local reactions

**Post-operative documentation**

**Surgical interventions and intra-operative diagnosis**

Surgical approach: dorsal/ventral/combined

Laminectomy: yes/no

Direct surgical reduction of retropulsed fragments of the posterior vertebral wall: yes/no

Corporectomy: yes/no

Surgical decompression: Level of spinal segments (f.e. T2 – T5)

Intra-operative findings: Opening of the dura: yes/no

Intra-operative complications

Implant-related revision surgery necessary: yes/no

**Post-operative imaging**

- Post-op CT and/or X-Ray and/or MRI (as part of standard clinical care)
- Restoration of alignment: yes/no
- Fracture morphology and instability criteria according the AOSpine classification

**Time sequence of acute care chain**

Accident / injury time (date, time)

Patient transport directly from the injury site to a study-center: yes/no

Admission to the emergency room (date, time)

Beginning of surgical intervention (skin incision) (date, time)

Duration of surgery (minutes)

Complete drug anamnesis: NSAID’s, antidepressants, anticonvulsants, opioids, spasmolytics, sedative-hypnotics, neuroleptics, anticoagulants (drug name and dosage for all)

Documentation of AEs and SAEs

**1. Follow-up exam, 24-72 hours post-operative**

Comprehensive medical history: etiology

Assessment of pre-existing neurological deficits

Revision and completion of time sequence of acute care chain (if not properly documented at baseline)

AIS/ISNCSCI

Plantar Reflex

Gait analysis

SCIM (Spinal Cord Independence Measure)

WISCI II (Walking Index for Spinal Cord Injury II)

CCI (Charlson Comorbidity Index)

ISS (Injury Severity Score)

Urological function

Drug anamnesis: NSAID’s, antidepressants, anticonvulsants, opioids, spasmolytics, sedative-hypnotics, neuroleptics, anticoagulants

Handing out patient diary (spasticity rating according to Penn Spasm Frequency Scale, subjective and objective other changes and special events)

Documentation of parameters from routine blood chemistry

Blood sample (only if baseline blood sample was collected): one serum tube (8ml)

Documentation of AEs and SAEs

**2. Follow-up exam, Day 14-21**

AIS/ISNCSCI

Plantar Reflex

Gait analysis

SCIM (Spinal Cord Independence Measure)

WISCI II (Walking Index for Spinal Cord Injury II)

Urological function

Retrospective assessment of physical activity prior to the injury

Drug anamnesis: NSAID’s, antidepressants, anticonvulsants, opioids, spasmolytics, sedative-hypnotics, neuroleptics, anticoagulants

Assessment of hand motor function in those patients who have lesions above T5

Differentiation: nerve root lesion vs. spinal cord lesion

Check patient diary

Documentation of parameters from routine blood chemistry

Blood sample (only if baseline blood sample was collected): one serum tube (8ml)

Planned inpatient rehabilitation: Where? When?

Documentation: date of discharge from the hospital

Documentation of AEs and SAEs

**3. Follow-up exam, after 3 months +/-2 weeks**

Patients who undergo rehabilitation in one of the three participating rehabilitation facilities in Austria have their follow-up exams there. All other patients are invited to the primary care center in which they received their initial treatment.

AIS/ISNCSCI

Plantar Reflex

Gait analysis

SCIM (Spinal Cord Independence Measure)

WISCI II (Walking Index for Spinal Cord Injury II)

Urological function

Drug anamnesis: NSAID’s, antidepressants, anticonvulsants, opioids, spasmolytics, sedative-hypnotics, neuroleptics, anticoagulants

Assessment of hand motor function in those patients who have lesions above T5

Differentiation: nerve root lesion vs. spinal cord lesion

Check patient diary

Documentation of parameters from routine blood chemistry

Blood sample (only if baseline blood sample was collected): one serum tube (8ml)

**4. Follow-up exam, after 6 months +/-2 weeks**

AIS/ISNCSCI

Gait analysis

SCIM (Spinal Cord Independence Measure)

WISCI II (Walking Index for Spinal Cord Injury II)

Urological function

Stability of osteosynthesis according to X-Ray/CT

Loosening of osteosynthetic implants? yes/no

Revision surgery necessary? yes/no

Drug anamnesis: NSAID’s, antidepressants, anticonvulsants, opioids, spasmolytics, sedative-hypnotics, neuroleptics, anticoagulants

Assessment of hand motor function in those patients who have lesions above T5

Differentiation: nerve root lesion vs. spinal cord lesion

Handing in patient diary

Blood sample (only if baseline blood sample was collected): one serum tube (8ml)

**Detailed Study Assessments**

**Urological function**

- Permanent catheter: yes/no
- Sensation of urinary bladder filling: yes/no
- Documentation of the first attempt of bladder emptying: pos/neg, date
- Self-catheterization: yes/no
- Do you feel sensory innervation of the external genitalia (penis / labia)?
- Do you feel the change of the catheter or manipulations on the catheter?
- Do you feel the urge to defecate?
- Do you feel stool evacuation?
- Male patients: Have you had an erection since your injury?
- Female patients: Have you felt sexually aroused since your injury?

**Hand motor function**

- Nine-Hole Peg Test (NHPT) (if feasible)
- Grasp and Release Test (GRT)
- Additionally, the following grasp tasks are recorded:
- Pinch grip: yes/no
- Clenched grip: yes/no
- Pencil grip: yes/no
- Lumbrical grip: yes/no
