# Supplementary material for: The effect of extracorporeal shock wave therapy in acute traumatic spinal cord injury on motor and sensory function within 6 months post-injury: a study protocol for a two-arm three-stage adaptive, prospective, multi-center, randomized, blinded, placebo-controlled clinical trial
Source: Trials. 2022 Apr 1;23:245. doi: 10.1186/s13063-022-06161-8 (PMC8973563; doi:10.1186/s13063-022-06161-8)
Supplement: Supplementary file 7 — Additional file 7. Investigational Medical Device (IMD) [file 13063_2022_6161_MOESM7_ESM.docx]

**Investigational Medical Device (IMD)**

**Name and Description of Investigational Medical Device**

The Medical Device used in this trial is a shockwave generator produced by MTS Medical UG, 78467 Konstanz, Germany. The orthogold100® uses patented MTS Spark Wave® technology.

The shockwave generator orthogold 100® generates high-energy acoustic waves that behave much like other sound waves except that they have much greater pressure and energy. As with sound waves, Spark Waves® can easily travel great distance as long as the acoustic impedance stays the same. However, when the acoustic impedance changes, energy is released; the greater the change in impedance the greater the release of energy. There is a much higher release of energy at the soft tissue/bone interface than at a muscle/fascia interface. The release of energy from the Spark Wave® within the region of the affected tissues and the resultant compression and tension of cells creates a positive physiological effect. Mechanotransduction is the physiological effect thought to be responsible for stimulating normal and injured cells to produce healing factors.

**Applicators**

The orthogold100® is available with various applicator therapy heads, providing a range of penetration depth, energy and focal size. Probe selection can be determined with the aid of ultrasound to measure depth of the intended target. For this trial the applicator OE050 will be used.

**Known Side effects**

No serious side effects have been reported by clinicians even when using highest energy settings, however the following minor side effects have been observed in isolated cases:

- Minor petechial bleedings may occur if the coupling between the probe cushion and skin is not air exclusive
- Occasional soft tissue swellings over treated tendons
- Pulmonary tissue tearing and extra-systoles
- Some patients experience a three to four day period of incomplete and transient pain reduction after ESWT
- Numbness over the treated area

No correlation to outcome or future responses to therapy has been established in cases where soft tissues swelling occurs. Aiming at pulmonary tissues or the trachea should be avoided.

The result of ESWT is not analgesia, but rather pain reduction. During this period it is important that patients rest in order to avoid over-working an injury thus risking re-injury. This should be taken onto consideration prior to performing ESWT on an athlete.

**Contraindications**

Below you will find the listed contraindications according to the manufacturer of the Investigational Medical Device.

- Do not use the orthogold100® in patients with pacemakers or implantable defibrillators.
- Do not use the orthogold100® in patients who are using devices which are sensitive to electromagnetic radiation.
- Do not use the orthogold100® in confirmed or suspected pregnancy.
- Do not adjust the therapy focus on internal organs (especially lungs).
- Do not use the orthogold100® for the treatment of patients with tumours.
- Do not use the orthogold100® for the treatment of patients with severe coagulation disorders.
- Do not use the orthogold100® for extracorporeal shock wave lithotripsy.
- Do not use the orthogold100® for the treatment of patients younger than 18 years or of patients with open epiphyseal plates.
- Do not direct the shock waves on large vessels.
- Do not direct the shock waves on internal airfilled organs (especially lungs).
- All other Contraindications mentioned in scientific literature

Due to our new scientific approach to use shockwaves in spinal cord injuries, some of the contraindications are not applicable for this study:

- Do not use the orthogold100® for the treatment of vertebrae, skull bones and ribs.
- Do not direct the shock waves on large nerves.
